# Teaching Digital Electronics during the COVID-19 Pandemic via a Remote Lab

**DOI:** 10.3390/s22186944

**Published:** 2022-09-14

**Authors:** Felipe Valencia de Almeida, Victor Takashi Hayashi, Reginaldo Arakaki, Edson Midorikawa, Sérgio de Mello Canovas, Paulo Sergio Cugnasca, Pedro Luiz Pizzigatti Corrêa

**Affiliations:** Polytechnic School, University of São Paulo, Luciano Gualberto, São Paulo 05508-010, Brazil

**Keywords:** distance learning, digital systems, internet of things, remote lab

## Abstract

Practical knowledge is essential for engineering education. With the COVID-19 pandemic, new challenges have arisen for remote practical learning (e.g., collaborations/experimentations with real equipment when face-to-face offerings are not possible). In this context, LabEAD is a remote lab project that aims to provide practical knowledge learning opportunities for Brazilian engineering students. This article describes how engineering project management methods consisting of application domains, requirement identification, technical solution specification, implementation, and delivery phases, were applied to the development of an Internet of Things (IoT) remote lab architecture. The distributed computing environment allows integration between students’ smartphones and IoT devices deployed in campus labs and in student residences. The code is open-source for facilitated replication and reuse, and the remote lab was built in six months to enable six experiments for the digital electronics lab during the COVID-19 pandemic, covering all the experiments of the original face-to-face offering. More than 70% of the 32 students preferred remote labs over simulations, and only 2 were not approved in the digital electronics course offered remotely.Student perceptions collected by questionnaires showed that they could successfully specify, develop, and present their projects using the remote lab infrastructure in four weeks.

## 1. Introduction

If engineers manipulate materials, energy, and information to create benefits for humankind, the application-based education must go beyond theoretical understanding and be combined with practical knowledge. Therefore, engineering programs must support the use of instruments and the validation of theoretical models in practice. This would allow students to develop data analyses, communications, and teamwork skills, and allow students to gain experiences from failures [1].

There are many online collaboration tools to use for remote learning; however, novel challenges arise in remote practical engineering education. Online labs can enhance remote learning in the science and engineering field by providing remote learners with hands-on experience [2]. There are two main alternatives for online labs: simulation labs and remote labs.

Simulation labs are built using simulators, which are used in many industries. Their advantages are portability, cost-effectiveness, and ease of use. However, practical knowledge obtained in simulation environments is limited to the capabilities of the software used [1].

Remote labs are used by academic researchers and students to perform laboratory experiments through the internet. A remote lab reduces the cost of running experiments by optimizing the sharing of lab resources, eliminating the need for physical presence, and promoting self-learning. Benchmarks found in the literature highlight common functionalities for remote labs: collaboration, video streaming to monitor real equipment, and scheduling to prevent conflicts [3]. The main advantages of remote labs are related to flexibility: if combined with an open lab paradigm, they allow students to perform experiments anytime, making the labs accessible from anywhere [1]. One interesting remote lab feature is collaboration: many students at different locations can work together to perform a lab experiment by using a remote lab tool [3].

Another feature involves the digital transformation that will occur in the industries that students will be a part of in the future. Digital transformation can be understood as the “*integration of digital technology into all areas of a business, fundamentally changing how you operate and deliver value to customers*” [4]. Additionally, digital transformation may be considered a survival tactic and a necessary long-term strategy for companies. Current and emerging technologies have enormous impacts on business processes and business models; therefore, deep knowledge and experience are necessary for these emerging technologies [5].

As no company can grow without innovation, and innovation depends on emerging technologies, technology teams are instruments of innovation and digital transformation in companies. Professionals must be able to track, pilot, and deploy technologies that impact their businesses (e.g., reduce costs or increase revenue) [6]. Considering the demand for emerging technological knowledge, we consider that students may be motivated and engaged in efforts that might be useful for the ongoing digital transformation.

The digital electronics lab discipline comprises about 15 classes of 3 h 40 min each, grouped in two phases: first, fundamentals regarding IoT platforms are presented to teach students on how to interact with the remote lab and FPGA (field programmable gate array) board and measurement tools, such as Analog Discovery devices. Some sensors deal with distance and sonar; some actuators, such as servo-motors, are applied to connect analog signals mapped to digital signals. Moreover, serial communication using the RS232C protocol to create a UART (universal asynchronous receiver–transmitter) component helps students to learn basic concepts about serial communication broadly used in most current digital types of equipment. The second phase is the project created by pairs of students. Each project has to include previous circuits: FPGA, UART, sensors, and actuators, all of them connected with smartphones and laptops (and occasionally with circuits implemented in their homes).

Each class occurs in a synchronous session that occurs as follows:

1. The teacher begins the class with a video conference tool and performs an initial student activity: a quick test regarding the experiment or a 3-min pitch of the current status of the projects. The teacher can also perform a short demonstration of the working experiment using the remote lab (15 min).
2. The students are separated into groups of two or three students each. This group organization is performed by the free choices of the students and this set is maintained in all classes and the project (10 min).
3. The students perform the practical activities using the remote lab. The teachers and students access the lab bench remotely, but technical staff are present in the university lab to perform maintenance (e.g., shutdown) during the experiments (3 h),
4. The teacher ends the group rooms and presents the instructions for the next experiment (15 min).

Moreover, the students who had problems in the class or desired to use the remote lab infrastructure for the following week’s assignment were able to schedule an open lab session with the lab’s technical staff.

Before the pandemic, everything in this lab was in a physical mode, i.e., students, professors, support specialists, and all-around lab devices were in a single place. With the COVID-19 pandemic context, this alternative has disappeared. At the start of the period, students used digital simulators to learn about circuits. That was just half of the learning cycle because there was no practical implementation or delivery, which frustrated all stakeholders (students, professors, and technicians).

In this context, LabEAD is a remote lab project meant to provide practical knowledge learning opportunities for Brazilian engineering students during the COVID-19 pandemic. The project is a collaboration between the University of São Paulo and the Federal University of ABC; it started in March 2020.

We developed LabEAD modules based on Arduino to make them accessible and easily reproducible. The low-cost hardware made it possible for students to adopt HomeLab kits, which are lab kits that allow students to perform lab activities at home. Although this idea is not new [7], to our knowledge, its integration into a remote lab course for student projects is novel. Another contribution regards the Internet of Things domain. As there are no standards or common definitions of the IoT, there are a lack of important metrics, such as user satisfaction, convenience, and safety [8]. The literature does not have a definition on the quality of experience in IoT, either, even though aspects such as availability, efficiency, and reliability have played important roles in recent discussions [9]. We do not intend to answer all of these questions about IoT, but we built the LabEAD architecture considering the distributed computing nature of IoT, combined it with software engineering quality methods, and made it available to students in digital electronic remote experiments during the COVID-19 pandemic. The projects idealized by these future engineers (using an entrepreneurship approach) are inherently based on IoT architectures, and they offer experimental starting points to address the open questions still present in the IoT domain.

Compared to previous research [10], which described the Analog electronics lab at the Federal University of ABC (UFABC), this article describes a case study for the digital electronics lab with student perceptions and performances at the University of São Paulo (USP).

The following research questions apply to this work:

**Research Question 1**: is it possible to create a fully remote course on digital electronics using IoT infrastructure? The student performance must be comparable between the remote and face-to-face offerings.

**Research Question 2**: do most students prefer the remote lab approach over simulations?

**Research Question 3**: can students use the IoT remote lab infrastructure to properly specify, develop, and present projects during the four-week course?

The text is organized as follows: Section 2 describes the related work found in the literature; Section 3 contains the engineering method and the IoT architecture of the remote lab used in the course. Section 4 presents the results: student performances, student perceptions, full description of one project, and comparisons with related work. Section 5 concludes the article with final considerations.

## 2. Related Work

In this section, the related works regarding remote and virtual labs found in the literature are presented.

The MASTERS VLAB (multimedia systems in telecommunications, medical, and remote sensing applications virtual lab) project supported 20 experiments for digital signal processing. Perceptions were obtained via a questionnaire with 31 students, and the subjective assessments indicated that the tool enhanced students’ understanding of the course material and that the interfaces were considered user-friendly. It was implemented in MATLAB and C++ and is freely available [11].

V-Lab is a cloud-based virtual lab that uses open-source virtualization techniques and software-defined networking solutions for network security education. V-Lab supported more than 1000 students from 6 courses, with over 20 experiments. It was evaluated with three surveys, with participation between 17% and 96% for 212 students. The results indicate that V-Lab reduced training hours, resulted in a higher completion rate, and that students that performed better in progressive experiments also achieved better grades with 99% confidence [12].

The Virtual Labs Open Educational Resources (VLAB OER) consortium was created with 12 engineering education institutions in India, with all content available under a Creative Commons license. A total of 1650 different experiments from 9 disciplines were chosen, and VLAB OER obtained more than 19,000 registered users from 2011 to 2013. Pre- and post-tests were applied to study the adoption of virtual labs, and the results showed that the following attributes—compatibility, ease of use, relative advantages, and trialability—explained 50% of the variations in the virtual lab adoption. Ease of use was the most dominant predictor. Overall, student feedback was positive, and student performances showed similar performances in the physical and virtual labs, with an average time of 50 minutes for virtual labs and 105 minutes for physical labs [13].

OPTILAB is a virtual lab for optics experiments built in Java. It was evaluated with 70 out of 83 students via the technology acceptance model method, with a control set of 44 students with an average score of 67.4% in the final exam, and an experimental set of 26 students with an average score of 49.2% in the final exam. Perceived ease of use had a positive effect on perceived usefulness, which had a positive impact on behavioral intention, as the more the user perceived the usefulness of the virtual lab, the higher the intention to use it. Behavioral intention also has a positive impact on the actual system use [14].

The remote lab Open Network Laboratory (ONL) was used in a graduate computer network course to provide remote access to experimental facilities with real systems for four experiments. The author evaluated the effectiveness of ONL in student learning with 6 assessments and 29 students; student participation varied from 31% to 93%. The contribution of the lab experience to student learning was comparable to lecture learning; hence, one major observation is that lab components were almost equally as important as lectures [15].

VISIR is one of the most popular laboratories for analog electronics experiments. A study with 159 students from 2 different academic years in a physics course investigated the effect of VISIR on students learning Ohm’s Law. The results with 141 participants showed that the use of remote labs had a positive effect on student learning, regardless of the previous practical experience [16].

The Advanced Digital Lab based on the MIT iLab architecture was used to allow students to implement and test finite-state machines in field programmable gate array (FPGA) devices. The study used questionnaires and tests applied to 510 students in 2012 and 2014, showing that remote labs can be almost as effective as traditional labs (concerning students learning advanced digital concepts). The study shows that remote labs cost less than traditional labs, and that remote labs do not compete directly with traditional labs, but rather complement them by offering more access [17].

Another study on undergraduate digital circuit learning was performed to compare face-to-face and online offerings. A live version was offered to 48 students in 2009 and an online version was offered to 48 students in 2010. The results show no noticeable difference between the two approaches [18].

A hybrid approach to teaching 802.11 network security (combining virtual and physical labs) was proposed as a safe playground for experimentation regarding attacking strategies. It was evaluated with 76 students over 4 semesters in a security concepts course, and its results confirm that the hybrid lab is as effective as a traditional physical lab with the advantage of enabling learning in co-located and online environments [19].

Mohsen et al. proposed a remote FPGA lab with a scheduling system and support to multiple concurrent users. It was validated with 50 undergraduate students with 3 experiments. No discussions on learning aspects were provided and it was not open-source [20].

An IoT remote lab was proposed to be used in lectures and projects [21]. It was validated with two applications used in lectures on “Computer Science” and “Big Data”, with no mention as to how many students used the tool. The solution enables real data collection and analysis using Simulink (part of MATLAB). Therefore, it is not open-source. Finally, even though the usage of remote labs in student projects was proposed, it was not achieved.

Another FPGA lab was proposed by Peinado et al. [22]. The authors used open-source to develop the web application that enabled remote access to the hardware board, but the solution was not provided under an open-source license. No learning outcomes were discussed and the solution was used by a small group of students with a focus on collecting technical metrics (e.g., resource access, demands, peripheral emulation).

An Arduino-based remote lab was proposed by Martin et al. [23]. It was validated with 12 experiments. Even though the authors presented the potential competencies of the students using the tool, no student perceptions or usage results were presented. It is not distributed under an open-source licence.

A FPGA remote lab was developed considering the students’ point of view for the interface design [24]. A sample experiment was presented; the tool was used by a class of students in 2018, and by users from 25 different countries from 2018 to mid-February 2019. However, although the remote lab is open for anyone to use, its code is not provided.

To the best of our knowledge, even though some solutions on remote labs for digital electronics courses could be found in the literature, none are open-source. Most of the related work used remote labs only for directed experiments (i.e., the students practice default experiments and do not use the infrastructure in projects).

## 3. Methods

### 3.1. Engineering Method

**Figure 1:**
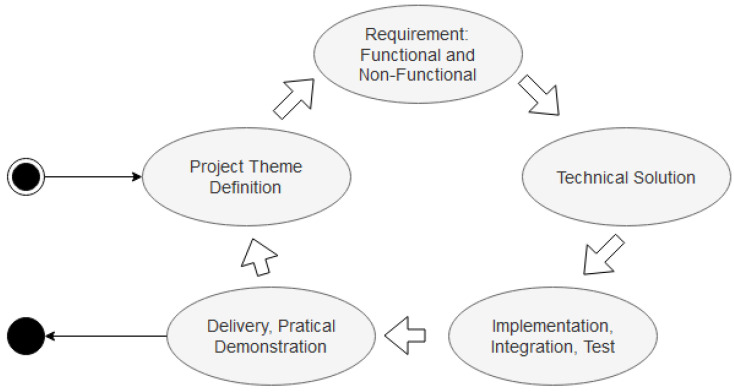
Project cycle: phases include quality aspects.

All lab activities were based on quality aspects to build digital systems, including digital circuits created on an FPGA board, IoT sensors, and actuators integrated into an ESP8266 module. The engineering method to build digital projects illustrated in Figure 1 is a guideline used for LabEAD development. The discipline creates activities focused on a primary engineering quality method that considers the following: definition of the application domain (or problem domain); identification of functional and non-functional requirements obtained by MVP (minimum viable product) agile techniques; technical solution phase, including hardware, software, internal and external integration aspects driven by test cases; and finally, delivery by project artifacts and a good demonstration of the built system’s dynamic behavior. In each phase, quality aspects were used as delivery drivers, as described in Table 1.

As mentioned, the discipline explores quality aspects in all phases of a project’s cycle. In the lab, some of the quality configurations applied are described in Table 1. It was essential for the student to learn that quality did not only regard implementation test cases and error debugging. What and how to test could not always be answered in the implementation phase. Students learned that such test cases had to be described early in the project cycle before they implemented the solution.

According to Table 1, the quality configuration parameters used by students were project theme definition, requirements elicitation, technical solution, implementation, and delivery.

In the project theme definition, quality parameters were considered in the scope of the project; students identified themes and adequate sizes, in terms of complexity and viability, using the MVP techniques, with the help of professors and the lab team. Some tools used in this phase were the Osterwalder Value Proposition Canvas [25] to make students think about the usefulness of the project; an example of a project scope definition is a decision regarding how many different modules the proposed architecture has.

Functional and non-functional requirements are defined and mapped with accepted test cases in the next phase. Such specification documents are input to organize the solution and plan tests and simulation sessions. The functional and non-functional requirements are defined with user journeys and ISO25010 [26], respectively.

The technical solution is organized as follows: parts of hardware, pieces of software. Two dimensions are necessary for the project. The first one, static, is related to the components and integration of digital circuits and software components. The second dimension refers to dynamic behavior, where inputs, outputs, integration, and signal/data transformation can execute the designed functionality when combined. A useful reference model for this step is the RM-ODP [27].

In the implementation phase, students learn that engineering activity is more than simply implementing and developing circuits or programs. They understand that quality can be obtained in all the activities of a project cycle, mostly when a system works correctly, after many error detection cycles, by applying test cases created in previous phases. For example, the students were encouraged to apply the agile development ideas [28] in this iterative process.

Finally, at the delivery phase, the discipline emphasizes the importance of preparing technical artifacts and evidences that the system is precisely built. Those artifacts demonstrate results and some limits and vulnerabilities. That is, professors can observe quality control aspects of the implemented system.

The lab resources include a number of tools and devices, but some of them particularly contribute to quality aspects: the virtual immersion involving students and professors involves guaranteeing the project scope from the very beginning to maintain all expectations at an adequate level (a); internet access to search and select information regarding problems and solutions from all over the world (b); the application of agile techniques to identify and present problem domains and requirements using videos and prototypes, instead of text documents (c); platforms and project tools, such as Quartus Prime [29], Analog Discovery [30], Anydesk [31], ModelSim module, Python language combined with remote connection provided by IoT ESP8266 modules, sensors and actuators, such as sonar and servomotors, Blynk IoT platform [32] to connect students to Android and iOS smartphones, and FPGA board in the campus lab (d). In this environment, the student can observe and measure input and output signals, the transformation of data by logical blocks, and he/she can also observe protocol data using the Analog Discovery tool to ensure the correctness of the results.

### 3.2. Internet of Things Architecture for Remote Labs

**Figure 2:**
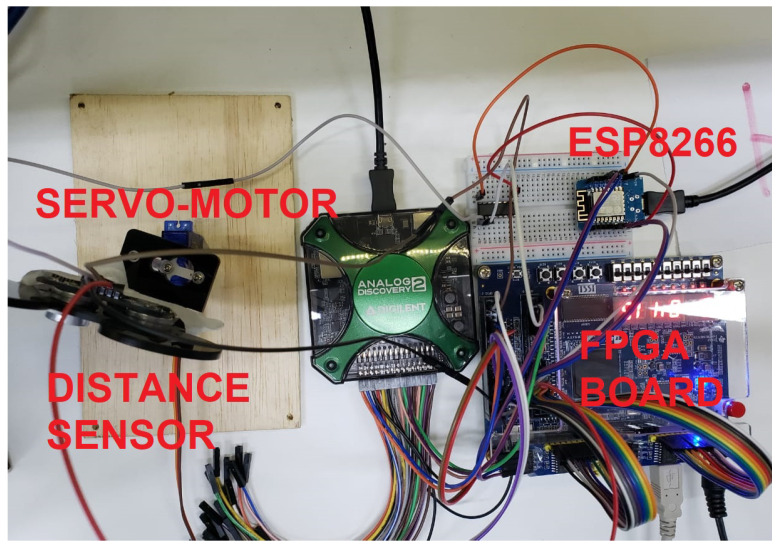
Lab bench with desktop, FPGA, and IoT devices.

The physical lab bench located in the discipline laboratory is presented in Figure 2. Each lab bench is equipped with electronic modules, sensors and actuators, increasing the possibility of conducting different experiments in the discipline. These components are connected to a FPGA board, which is the core of the discipline. Each student interacts remotely with the lab bench using an IoT platform. We opted to use the commercial product Blynk [32] as this platform allows communication between the student’s smartphone, an ESP8266 device (i.e., an intermediate between the FPGA and the Blynk Cloud Platform), and the desktop computer at the lab bench.

This communication takes place using an authentication token, which is a private identifier for the IoT platform. This token is generated when students create projects on their smartphones. It must be configured on the remote lab bench so that they can send commands to the lab bench using their smartphones. The Blynk platform allows students to create some visual user interface components in their projects. The components can be logically mapped into physical pins of the ESP8266 on the lab bench or virtually on the cloud server. Therefore, it is possible to remotely change the logical level of a pin by changing the state of a widget on the smartphone screen.

Communication of the ESP8266 with the smartphone is enabled using a library available in the Arduino IDE. This allows establishing a connection with the server using the authentication token configured in the ESP8266 sketch code. The communication between the desktop and the smartphone takes place with an interface programmed in Python, which receives commands from the student’s smartphone to perform corresponding operations on the remote lab bench. These commands are read through HTTP requests to the Blynk cloud server. Among them, the operations of compiling the digital system project, project programming, and tests on the FPGA board occur, allowing the student to perform the complete digital system development cycle.

During classes, the student remotely connects to the lab bench desktop computer using Anydesk. The connection is realized with the support of a lab technician that authorizes student access, monitors the student activity, and is responsible for terminating the session. Each lab bench has a webcam, which provides visual feedback, allowing the student to observe the status of the FPGA board, sensors, and actuators in real-time. The student is responsible for configuring his/her Blynk project authentication token in the Python script and then initializing it. After its initialization, a menu is opened containing several options that the student may select by using text-based commands given on his/her smartphone. Regarding communication, no considerable latency was observed throughout the course.

Throughout the COVID-19 pandemic, the technical network support team in the university was restricted to a minimum, so additional security mechanisms that required network configuration were not possible. However, the development team considered that even though the first version relied on manual access control and monitoring by the lab technician, with institutional support, it was possible that the next versions have automated security mechanisms. Such mechanisms could be found as suggestions in the literature [33]: integration with university single sign-on, restricted access to the remote lab from a specific virtual private network (VPN), and additional firewall rules configuration. Additionally, registering the users, their IP addresses and activities automatically in log files [34] can contribute to the ’rastreability’ of failures and misuses.

Students must configure the project file name in step 1, configure the file name containing the project pin designation in step 2, compile and program the project in steps 3 and 4, respectively. In step 5, students compile and program the INO script on ESP8266 with their token. Finally, they perform tests on the project in step 6.

The steps described previously were performed using corresponding mnemonics. The first two steps were responsible for setting the parameters used by the Python script. Steps 3 and 4 were related to the FPGA board development environment, in which our discipline used Quartus Prime 16.1. Step 5 used the Arduino command-line interface. Finally, in step 6, the input values of the circuit in the FPGA varied. This variation was possible by using connections between the ESP8266 device and the FPGA board, where the ESP8266 pins were connected to the GPIO pins on the FPGA board. In this case, commands made by the students changed the logical values of the ESP8266 pins, making changes to the inputs of the FPGA.

**Figure 3:**
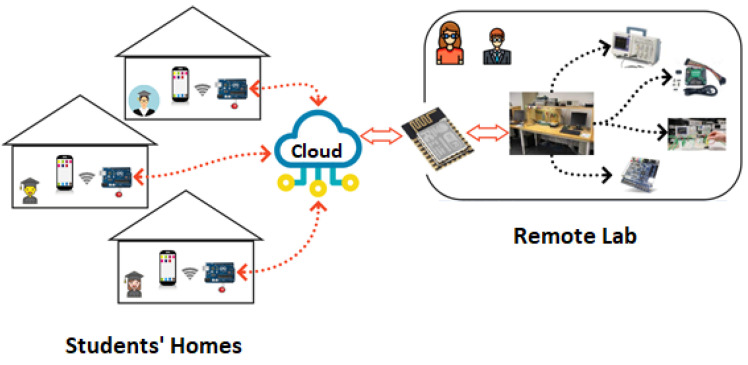
HomeLab integration via a remote lab.

In addition to the remote lab bench, the proposed architecture also allowed creating an environment called HomeLab, as shown in Figure 3. This environment was located in the student residences, where they could connect to the remote lab bench using another ESP8266 device, exchanging signals between their residence and the laboratory. This additional ESP8266 device ran a sketch that also connected to the Blynk IoT cloud platform. As an example, consider a situation in which students have a temperature and a humidity sensor at home. Data generated by these sensors can be sent to the laboratory by the ESP8266 from their home, allowing the FPGA to process these data. We understand the HomeLab as a way to empower students, allowing them to conduct different projects, and not limiting them to the infrastructure assembled on the remote lab bench.

In summary, the main opportunities associated with the HomeLab are to allow the students to integrate everyday elements, such as temperature sensors, motion sensors, images, or even actuator devices based on buttons and motors to enable the understanding of practical applications and projects with real-world scenarios. This possibility of applying knowledge to solve real-world problems is expected to increase student engagement. To our knowledge, using a HomeLab via a remote lab to close the gap between controlled lab environments and real-world scenarios has not been investigated in the literature [17,18,20,22,24].

The digital electronics lab course (learning objectives) concerned hardware design and implementation. This practical experience was reached using very high speed integrated circuit hardware description language (VHDL) and the FPGA board. Considering these course constraints, the HomeLab concept has become a set of complementary accessories that students could add to build interesting projects, but the project’s main functionalities must be implemented in the FPGA board. The evolution in the laboratory environment using HomeLab and a remote lab add opportunities in a complementary way, never replacing the laboratory environment with digital accessories that students can acquire and have at home, as it may seem at first glance.

Taking into account other participants, such as professors and technical assistants, it is possible to state that all of the participants greatly improved their knowledge, especially during this pandemic period. At the beginning of the pandemic, students worked with partial project cycles, i.e., they would use simulation tools to verify their projects. Then, they executed half of a project cycle without the implementation and delivery phases.

With the LabEAD remote lab, the results were fascinating: students integrated a remote lab with a HomeLab. By using IoT, remote sensors and actuators can be connected to home devices in real-time. It is the belief that new projects will emerge over this infrastructure lab, with new levels of applications and student experiences.

## 4. Discussion

### 4.1. Student Performances

We conducted a total of 6 experiments for 32 undergraduate students who worked in pairs during the practical activities. The digital electronics lab course ensures some technical skills, such as serial communication, interfacing with sensors and actuators, and that all projects and practical activities are backed up in the engineering method presented in Section 2.

**Figure 4:**
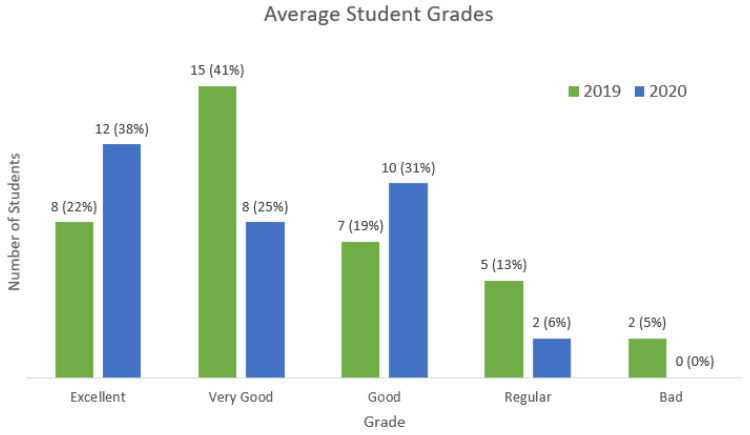
Comparison of face-to-face vs. remote offering.

In 2020 (face-to-face), all 32 students completed the course successfully with a final grade above 5/10 (i.e., requisite for approval), with an average student grade of 8.38, maximum of 9.87, and minimum 5.17. In 2019 (remote), 35 out of 37 students completed the course, with an average student grade of 7.89, maximum 9.52, and minimum 2.81. Figure 4 presents the average student grades classified in excellent (final grade above 9), very good (between 8 and 9), good (between 7 and 8), regular (between 5 and 7), and bad (less than 5) categories.

### 4.2. Student Perceptions

The student perceptions were collected with three questionnaires, with four to nine questions described below. The first survey was conducted at the beginning of the course, by the end of the first class, which was a “getting started” activity with the LabEAD remote lab. The second survey was conducted in the middle of the course, and the last survey was conducted at the end, when all the students successfully completed the course. The questionnaire is presented in Appendix A.

Considering that reliability and validity are defined as trustworthiness and rigor in qualitative research [35], and their relevance to eliminate researcher bias, a reliability analysis was performed.

The reliability assessment was based on Miles and Huberman [36] criteria, considering the applicable questions:

- **Question**: Are the research questions clear and are the features of the study design congruent with them?
  **Answer**: The three research questions were clearly defined. The first research question was investigated by comparing the student performances to evaluate if the digital electronics course using IoT could be delivered in a fully remote way. The second question was evaluated using a student survey. The third research question was analyzed based on a case study.
- **Question**: Were data collected across the full range of appropriate settings, times, respondents, and suggested research questions?
  **Answer**: Three questionnaires were applied to the students in the beginning, middle, and course ending. All students were invited to answer the surveys.
- **Question**: Were any forms of peer or colleague reviews in place?
  **Answer**: Three researchers were responsible for the questionnaire design and four researchers were responsible for peer review of the questions and student answers. Three researchers performed the analysis and colleague reviews of their (each other’s) analyses.

Key criteria to assess quality in qualitative research from the literature [37] were also considered. Regarding dependability, we considered that there was sufficient information for research replication (e.g., the remote lab was open-source and the research questions and approaches were clearly defined). Regarding confirmability, we used quotes and detailed descriptions to show how the findings were made.

Among the methods used for increasing validity [38], the prolonged engagement was applied, as the researchers were also the course’s technical assistants and, therefore, present throughout the whole process. We applied investigator triangulation and method triangulation (i.e., comparing the qualitative student perceptions and the quantitative student performances to answer the research questions).

Regarding participation, 25 out of the 32 students replied to the first questionnaire, 20 to the second, and 11 to the last one. All feedback was optional and anonymous, and each question had a space for free comments.

Regarding the first question, in the first survey, 10 out of 25 participants (40%) reported that they only knew the theoretical concepts of serial communication, actuators, and sensors, and the remaining 15 reported that they knew nothing (60%). In the last questionnaire, 11 participants reported that LabEAD allowed remotely learning these concepts. Previous knowledge was obtained in other theoretical disciplines, as explicitly stated in the following student comment: “*I know very little, just the content covered in previous disciplines in the course*”. Between the different concepts, one student said that the most relevant one was serial communication: “*This knowledge is important, especially the serial, which forms the basis of communication between components*”.

**Figure 5:**
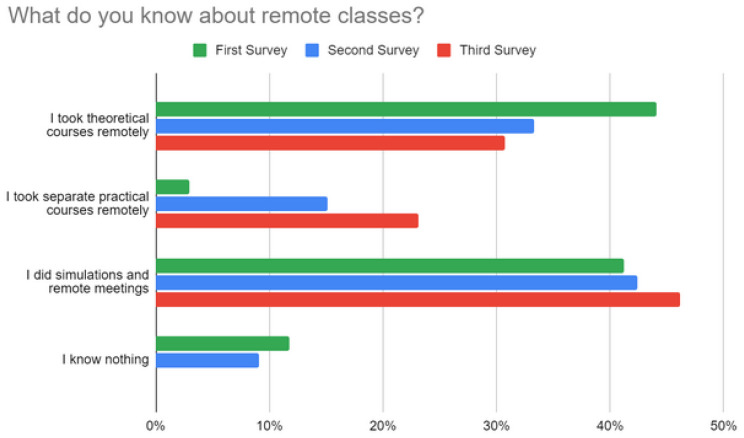
Remote class perceptions from students.

Most students took theoretical courses, and they performed simulations and meetings remotely. Only a few took practical courses remotely, which validates our initial assumption that the practical activities of these students during the pandemic were limited, as shown in Figure 5. One student stated that “*LabEAD contributes to a new vision of remote classes*”.

The initial perceptions of LabEAD were positive overall. Only 2 student out of 25 had problems and could not perform the activities and provided negative feedback. This occurred due to a problem with the university lab computer, which kept the machine in a constant cycle of rebooting; 84% of the students (21 out of 25) liked the possibility of connecting home and school, and 76% (19 out of 25) agreed that they understood the remote access. One relevant comment is: “*I would like to congratulate the teaching team on the activity prepared for the class. It was the best remote class experience I’ve had so far, even with a series of complications*”.

Using a virtual command terminal with a mobile interface was a proposal that 88% (22 out of 25) students liked. In this sense, the students provided constructive feedback such as “There were minor configuration issues (which could be resolved quite smoothly). There was a slight delay in the execution of the terminal commands, which raised doubts as to whether the commands were correctly executed” and “*I thought it was nice to use the FPGA through the terminal, it was a good experience, but I think I still prefer to have the virtual buttons/switches*”. Another student also commented on the IoT Platform usage with constructive feedback: “*At first I thought Blynk was unnecessary and I didn’t like it, but now I like it (especially when we use it to control the servo motor, it’s fun). Regarding the CLI (command-line interface) application, it could give more feedback on bugs and when things are in progress. It’s important to handle error cases, too. In the early classes, there were times when unhandled errors stopped the script from running*”.

**Figure 6:**
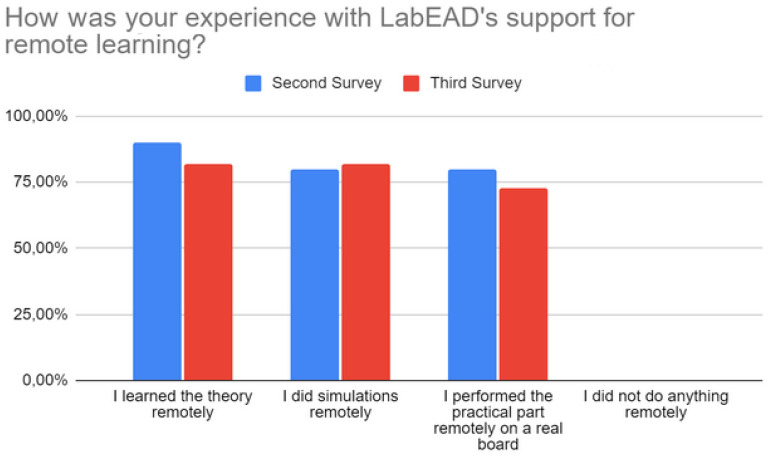
LabEAD support for remote learning as perceived by students.

Regarding the first additional question, 82% (9 out of 11) of the students agreed that they learned the practical part of serial transmission and reception, and 91% (10 out of 11) agreed that they learned a little regarding the remote control of a servo motor. The experience with LabEAD support for remote learning was also positive, with more than 70% of participants in the intermediate and final surveys agreeing that they could not only learn the theory and run simulations but also perform practical activities remotely, on a real board, as observed in Figure 6.

**Figure 7:**
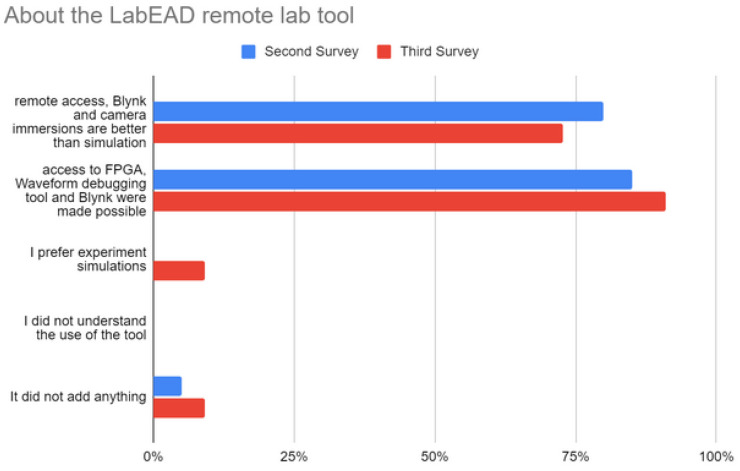
Student perceptions of the LabEAD tool.

Figure 7 shows that more than 70% of the students agreed that they preferred using remote access, the Blynk IoT platform, and a camera over simulations in the second and third surveys, with only one student in the third survey stating that the tool did not add anything and that simulations were preferred in his/her perception. Although simulations are equally important, we advocate that providing a remote lab with real board deployment and the usage of an IoT system may help students acquire skills that can be useful in real-world projects, such as identifying problems, proposing, and testing solutions based on the tests performed.

Regarding evaluation methods, most of the second survey participants agreed that the freedom supported by the project should foster creativity (60%, or 12 out of 20 students). However, most students also had doubts about the project scope, as it was not imposed, but defined by the students (55%, or 11 out of 20 students). Only 3 participants out of 20 preferred the directed experiments over the project, and no student preferred tests over projects.

**Figure 8:**
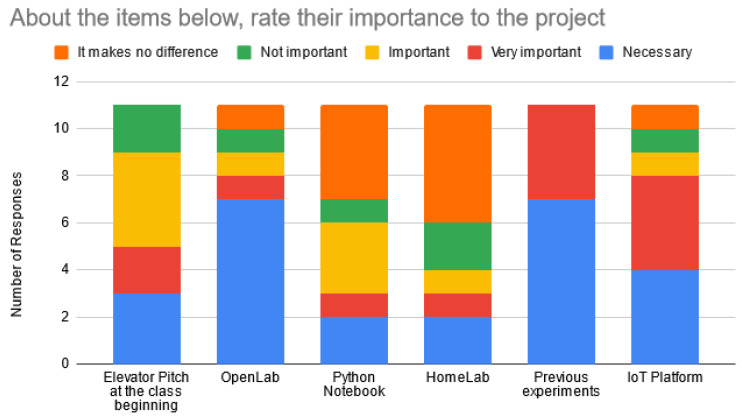
Item importance to the project.

Students considered the OpenLab sessions and the previous experiments as the most important elements to the project development. Other relevant elements were the IoT platform and the presentation performed at the beginning of the class, as shown in Figure 8.

**Figure 9:**
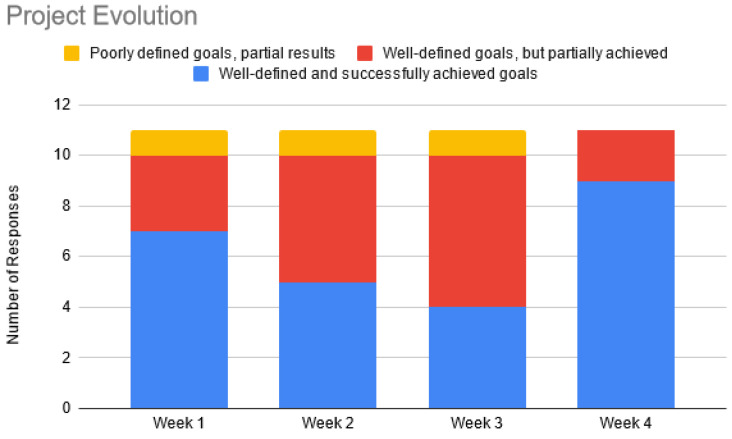
Project evolution in 4 weeks.

The trade-off between creativity and perceived uncertainty was further explored in Figure 9. Students considered that they could successfully define goals, with partial results in weeks 2 and 3. Most results were obtained at the end of the project, which is one aspect that could be improved in a sprint review for each team. One student commented on his/her project evolution: “*The goals of weeks 3 and 4 were well defined, however, due to technical problems, it was not possible to finish what was expected within the laboratory hours. These goals, however, were met during OpenLab hours. Furthermore, week 1 was planned, but in the middle of the development we thought it would be better to reorganize the ideas and plan in a more organized way*”.

**Figure 10:**
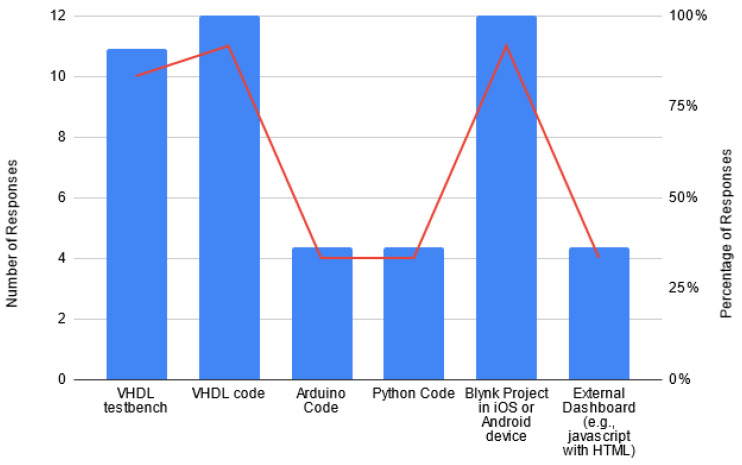
Artefacts developed by students in the projects.

As observed in Figure 10, all students used the mobile interface and developed the VHDL code for the FPGA board as represented by the blue bars, which indicate the percentage. Moreover, the red line indicates the absolute number of students who selected each option instead of the percent value. A total of 10 out of 11 students who participated in the third survey used the VHDL test benches for the simulations, and 4 developed the Arduino code (HomeLab), Python code and External Dashboard.

Regarding some suggestions that the students provided (if they were to develop the project again), some suggestions related to technical issues: “*If the project were conducted again, the group would probably have made a slightly more elaborate dashboard (using a specific tool such as React)*”, “*I would have defined an initial dashboard, before integrating with everything to identify what values I would like to present*”, “*I would have thought better of how to do the physical implementation*”, “*I believe the group could have started studying the Blynk platform earlier*”.

Additional suggestions regarded the project organization: “*I would have planned the first week better, so that we wouldn’t have wasted a week to reorganize the project and we would have already implemented more components for the second week*”, “*Better planning, mainly in defining the scope, so as not to have scalability issues and features that break the previous code*”.

Some students reported that they could learn technical concepts: “Consolidation of serial communication concepts, testing circuits with test bench, integration of modules to compose a complex system, remote communication with digital circuits (ESP and Blynk)”, “Serial and Blynk communication”.

Other students considered that project organization, collaboration, and communication skills could be learned: “*Work organization, preparation and development of the laboratory, understanding a problem and a project*”, “*Teamwork, IoT, organization and planning of tests, idea development, presentation in pitch format with limited time*”, “*Pitch elaboration, as well as improvements in error-finding strategies and improvements in planning skills*”. Ten out of 11 students evaluated their remote collaboration in the groups of two with the highest grade (5).

This combination of technical and other skills can be observed in the following student’s comment regarding his/her learning during the course: “*A first point is the definition of tasks and organization of teamwork. In addition, a great deal of knowledge about the functioning of IoT equipment was acquired with the Blynk and LabEAD equipment. Finally, knowledge about serialization, sensors and actuators was effectively acquired, both in the directed classes and for the development of the project*”.

### 4.3. Student Project Description

In this section, we present one student’s project description in detail. This project was developed by a pair of students using the remote lab infrastructure. The students conducted the complete project from problem definition, specification, planning, development, and documentation under the teaching staff guidance. The students could choose the project theme under the restriction that it used the remote lab infrastructure.

The student project described in this section was the Smart Trash Bin. Other examples of the 15 projects that the 32 students developed using the remote lab infrastructure were:

- Automatic alcohol gel dispenser;
- Smart watering for smart homes;
- Indoor safety for puppies;
- Vacancy sensor for parking lot;
- Smart door for smart homes.

The main objective of the Smart Trash Bin project was to understand the problem of solid waste in the world and, specifically, in the city of Sāo Paulo, and from then on to propose a solution that used the components developed in the Digital Laboratory II discipline in 2020 (i.e., UART, Distance Sensor and Servo-motor). At the end of the project, the team should have been able understand how the structuring of a project worked; being addressed: definition of requirements, division of tasks by components and by time, project understanding, construction of solutions in addition to validating the proposed project.

#### 4.3.1. Introduction

Cities are increasingly developing. Regulatory organizations and the population itself have been demanding intelligent management that makes strategic use of infrastructure, services, information, and communication to respond effectively and efficiently to the social and economic needs of cities.

In the Sāo Paulo city in Brazil, an average of 18,000 tons of garbage is generated every day. This is equivalent to 6.5 billion kilos of waste collected in a year by this city alone. In 2012, the capital’s monthly waste management was close to BRL 56 million (Brazilian Reais—BRL); that is, an expense of BRL 672 M per year. The recycling process in Sweden was the inspiration for the project.

#### 4.3.2. Specification

To develop the technical solution, we list the main functionalities of the smart recycling bin:

- Checking the amount of space available in the smart recycle bin;
- Sending the amount of waste present in the smart bin to the central;
- Opening the gates of the smart bin, driven by the truck;
- Sending the current conditions of the trash, indicating how much space there is still in the trash can. When requested by the central, by sending the character I.

We also list the main control center features:

- Checking the recycle bin status;
- Presentation of the status of each dump present in the project addressed by the central. Separating into status: normal, intermediate, and critical.

#### 4.3.3. Planning

In the first week, all the components were refactored. The adaptation of some components was performed in week 2, and the integration tests with iterative modifications happened during week 3. The presentation and documentation were executed in week 4. Each week, a short two-minute oral pitch was presented by the students at the beginning of the class with the current status and planned activities.

#### 4.3.4. Development

The servomotor is responsible for opening the dump gates when the truck is near, and closing them when the truck finishes emptying it. For this, we modified the component to have only two positions: one that indicates that the gate is closed, zero degrees, and one that indicates that the gate is open, 90 degrees.

The distance detector sensor is responsible for checking the capacity remaining in the smart bin. As we are still dealing with a test environment, we decided to adopt the total length of the recycle bin as 50 cm, with the critical state being triggered when the recycle bin is less than 10 cm in space. The necessary modifications for this component is the creation of a warning sign when the component has less than 10 cm of space.

The UART is responsible for communicating with the control center. It should send the trash status, represented by the characters C or N. The UART is also responsible for receiving the information request from the control center, represented by character I, and returning the space value still remaining in the recycle bin (represented by numeric characters). After receiving the data, the control center will release an R signal indicating the receipt of the data. The UART component did not need any changes.

The students chose to create a central that controlled eight trash cans; however, we left the implementation with a generic constant, which allowed us to easily change this number. The control center must check all bins in order and, if one of them is in critical condition, it must send the bin number followed by the distance from the bin to the bin lid to the Blynk terminal, allowing the central to take decisions. The students developed the control unit and the data flow of this central.

**Figure 11:**
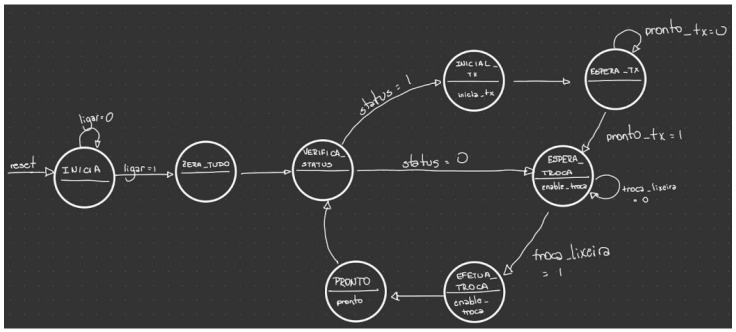
Finite state machine of the control component.

For the control unit illustrated in Figure 11, the students developed a state machine that checks if the selected bin is in critical status, if it is, the machine must send the value of the bin distance to the Blynk terminal. If not, it forwards directly to the “Wait for Exchange” state, which then forwards to the “Execute Exchange”, which tells the data flow to select the next dump.

**Figure 12:**
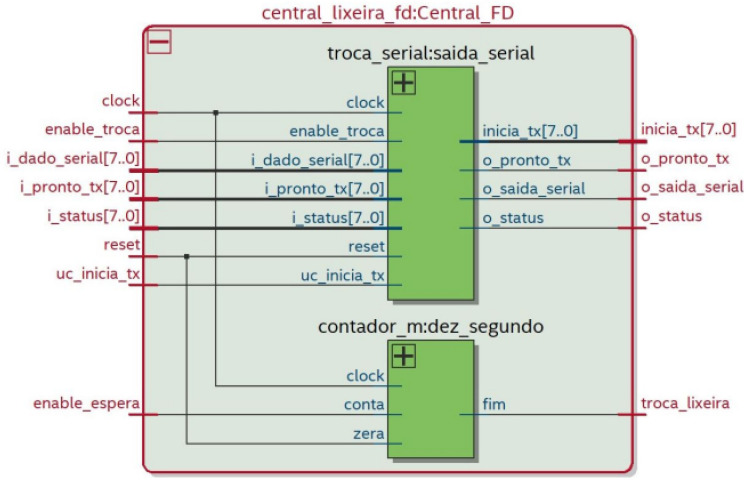
Data flow of the control component.

The central data flow depicted in Figure 12 has two components: the serial exchange and M Counter. The first one has the function of receiving the data: ProntoTX, serial data, and status of all recycle bins and it must control which recycle bin will be selected and which data will be highlighted. The M counter will wait ten seconds to move to the next bin.

#### 4.3.5. Validation

The students planned and performed the validation of each component (e.g., servomotor, distance sensor, serial communication). The integration test was executed with an automated test bench developed by the students.

In principle, the status was received by a recycling bin, which is the information that will tell the control center whether or not it is in a critical state. After receiving this information, if the status is ’, the control center sends data Initia_TX in which it starts the serial transmission of that data to the control center. After receiving the data, the recycle bin returns that it received the data correctly and continues operating and checking the operation of other recycle bins.

**Figure 13:**
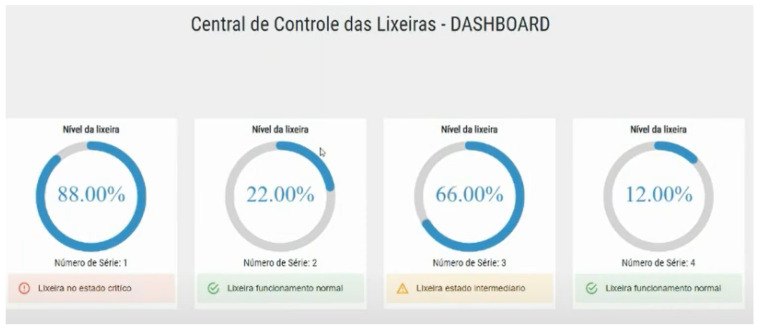
Smart Trash Bin dashboard.

A web dashboard integrated with the Blynk HTTP communication was also implemented by the students. In the final presentation, the dashboard illustrated in Figure 13 presented the remote lab prototype status in real time. The other two trash bins were simulated with periodic updates to show if the control component was operating successfully.

#### 4.3.6. Students’ Considerations

Throughout the development of the project, the pair managed to improve skills that had been worked on throughout the graduation course, such as communication (including communication with the Digital Laboratory team and colleagues); through presentations, planning, through organization, time, and coding, among others. The project also allowed the pair to consolidate teachings learned during the two Digital Laboratory subjects, such as componentization, project architecture (divided into data flow and control unit), component and project simulations, as well as physical and general project organization. In addition, this year, we had the opportunity to work in the EAD laboratory, which allowed the members to use technology used at a distance for the first time.

### 4.4. Comparison with Related Work

Some related works regarding simulation labs, remote labs, and hybrid labs found in the literature are presented next. The related works are summarized and compared with LabEAD in Table 2.

LabEAD is a remote lab that supported six experiments for the digital electronics course, supporting the education of 30 future engineers during the COVID-19 pandemic, considering only 2 out of 32 students dropped out of the course in the 2020 course offering.

Moreover, LabEAD also supported the following digital electronics course offerings in 2021 and 2022. Our remote lab supported a total of 134 students during the COVID-19 pandemic: 32 students in 2020, 41 students in 2021, and 61 students in 2022.

Although the total number of experiments and students can be considered low when compared to the related work, we must consider that LabEAD was quickly built to tackle the disruptive turn from face-to-face to remote offering in a matter of six months: from March to August, with the course offered from September to December. Furthermore, the team had no prior experience with remote labs.

Another relevant considerations regard the availability of the remote lab source code: it is open-source to foster reuse and collaboration between engineering institutions. If there was one lesson learned in this pandemic, it is that we must collaborate in times of need. It is available under a Creative Commons license in the following GitHub repository:

- https://github.com/vthayashi/labead-labdig (accessed on 12 April 2022)

To the best of the authors’ knowledge, this work is one of the first to present a remote lab for digital electronics with the results regarding student perceptions and with the benefit of a distribution of the code under an open-source license.

When compared to the related work, one key difference is that the remote lab infrastructure was used by the students in their projects in addition to the directed experiments. There are some related work with directed experiments using a remote lab for digital electronics [20,23,24]. These experiments were conducted in controlled environments with pre-visible results. Thus, a limitation we found in the state-of-the-art is that there were no reports regarding using a remote lab for student projects with flexible architecture and open experiments to foster student creativity. LabEAD supported the student projects in the second half of the digital electronics course.

## 5. Conclusions

Regarding the Research Questions, all of them could be answered based on our findings:

**Research Question 1**: It was possible to create a fully remote digital electronics lab course using the IoT infrastructure (i.e., Blynk). All the six experiments from the original face-to-face offering could be executed in a fully remote way. Student performances were slightly better in the remote offering (2020) compared to the previous face-to-face offering (2019). The remote lab supported the education of 134 computer engineering students.

**Research Question 2**: More than 70% of the 32 students from the 2020 course offering preferred remote lab over simulations. These results should be treated as a case study and future work may address the statistical relevance of such a comparison.

**Research Question 3**: The students could use the IoT remote lab infrastructure to properly specify, develop, and present projects in the four-week course in teams of two members each. The Smart Trash Bin project (2020) is a case study presented in this text that supports this statement.

Instead of a half-cycle project, the pandemic period inspired the teaching team and students to improve the use of remote, virtual, and immersion techniques based on IoT infrastructure. Some other results related to digital transformation in society are innovation and startup initiatives. All the phases of a student’s project combine agile rituals and presentation speeches in start-ups pitches known as "*elevator pitches*” [39]. In the end, in a virtual event, the students demonstrated their final projects to other professors, and competed for awards for the most valuable projects. In terms of technology, it is very attractive to students who configure hardware circuits using hardware languages with software tools available in the Python ecosystem, such as machine learning, algorithms, cloud Python notebooks, and data science libraries. All of this is connected to Android and iOS smartphones.

Student perceptions regarding the remote lab usage in upcoming offerings could be assessed using the User Experience Questionnaire for remote labs proposed by Cuadros et al. [40]. This was not feasible for this work because this questionnaire was published in 2021 and our in-depth evaluation was conducted in 2020.

Other opportunities are related to capstone projects. This lab is digitally connected and it allows for some digital improvements. Consider energy and devices control: new IoT functionality to control energy consumption, on/off period to adapt class periods and weekend days. Regarding access control, logging, and scheduling functionalities to monitor and enforce authorization rules to different user profiles are possible for future work. Open datasets for data science are other opportunities. All activities, codes, and execution events stored in a cloud environment could be used as learning data to improve new lab activities. All circuits and projects created by students could be used as catalog data in future projects by other groups of students. Finally, the results may be presented to digital companies to inspire industrial projects to improve digital services in various application fields. Moreover, companies may contact students and create information technology job opportunities for the most engaged students.

## Figures and Tables

**Table 1:** Quality aspects of project phases.

Phase	Quality Aspect
Project Theme, Application	MVP product, focusing on agility, contributing to small definition as a scope toward real delivery
Requirements: Functional and Non-functional	Direct mapping of functional and non-functional requirements as inputs of acceptance tests and correctness verification
Technical Solution	Organization of the system as a set of hardware and software components. Integration tests, unity tests to obtain quality, driven by the previous phases (requirements, verification, and test plans)
Implementation	The student produces a correct project by implementing hardware and software solutions step-by-step: implements, tests, integration, tests. Each right part acts as a stimulus to continue the project. Simulation is a powerful tool in this phase.
Delivery	Each tested part acts as a delivery. The students are encouraged to deliver and demonstrate correct details in an evolutionary delivery approach

**Table 2:** Comparison with Related Work.

Lab	Category	Subject	Number of Students	Number of Experiments	Learning Outcome	Open-Source
MASTERS VLAB [11].	Virtual Lab	Digital Signal Processing	31	20	The tool enhanced students’ understanding of the course material	yes
V-Lab [12].	Virtual Lab	Network Security	212	20	Students who performed better in progressive experiments also get better grades with 99% confidence	yes
VLAB OER [13].	Virtual Lab	9 Disciplines	19000	1650	Overall student feedback is positive, and student performances show similar performances in physical and virtual labs.	yes
OPTILAB [14].	Virtual Lab	Optics	83	N/A	N/A	no
ONL [15].	Remote Lab	Computer Networks	29	4	The contribution of the lab experience to student learning was comparable to lecture learning.	no
VISIR [16].	Remote Lab	Analog Electronics	159	N/A	The use of remote labs has a positive effect on student learning regardless of their previous practical experience	no
Advanced Digital Lab [17].	Remote Lab	Digital Electronics	510	N/A	Remote labs can be almost as effective as traditional labs considering student learning advanced digital concepts.	no
Undergraduate Digital Circuits Lab [18].	Remote Lab	Digital Electronics	96	N/A	The results show no noticeable difference between online and face-to-face approaches.	no
Hybrid 802.11 Network Security [19].	Hybrid Lab	Digital Electronics	76	N/A	Hybrid lab is as effective as a traditional physical lab.	no
Mohsen et al. [20].	Remote Lab	Digital Electronics	50	3	N/A	no
Leisenberg and Stepponat [21].	Remote Lab	Computer Science and Big Data	N/A	N/A	N/A	no
Oballe-Peinado et al. [22].	Remote Lab	Digital Electronics	N/A	N/A	N/A	no
Martin et al. [23].	Remote Lab	Digital Electronics	N/A	12	N/A	no
Schwandt and Winzker [24].	Remote Lab	Digital Electronics	N/A	1	N/A	no
**LabEAD**	**Remote Lab**	**Digital Electronics**	**134**	**6**	**The remote lab enabled all the face-to-face experiments during the pandemic, with overall positive student feedback**	**yes**

## Appendix A. Questionnaire

### Appendix A.1. First Survey

1. What do you know about serial digital communication, sensors, and actuators? (You may select more than one option).
  (a) Only theory
  (b) Technical course
  (c) Hardware and software internship
  (d) I know nothing
  (e) LabEAD allowed remote learning
2. What do you know about remote classes? (You may select more than one option).
  (a) I took theoretical courses remotely
  (b) I took separate practical courses remotely
  (c) I carried out simulations and remote meetings
  (d) I know nothing
3. About the first part of today’s workshop, turning on LED by cell phone (you may select more than one option):
  (a) I liked the possibility to connect home and school
  (b) I understood the remote access
  (c) I did not understand the IoT platform
  (d) I did not like it
4. About the second part of today’s workshop, interaction via the virtual terminal (you may select more than one option):
  (a) I have suggestions
  (b) I liked the proposal
  (c) I could only use remote terminal
  (d) I could only use remote access
  (e) I did not like it

### Appendix A.2. Second Survey

1. What do you know about serial digital communication, sensors, and actuators? (You may select more than one option).
  (a) I learned a little about the RS232C protocol
  (b) I learned the practical part of serial transmission and reception
  (c) I learned a little about remote control of a servo motor
  (d) I already knew about the technical course/internship
  (e) I did not learn anything
2. How was your experience with LabEAD support for remote learning? (You can select more than one option).
  (a) I learned the theory remotely
  (b) I carried out simulations remotely
  (c) I performed the practical part remotely on a real board
  (d) I did not do anything remotely
3. About the LabEAD remote lab tool (you may select more than one option):
  (a) Remote access, Blynk and camera immersions are better than simulation
  (b) Access to FPGA, Waveform debugging tool and Blynk were made possible
  (c) I prefer to simulate experiments
  (d) I did not understand the use of the tool
  (e) It did not add anything

### Appendix A.3. Third Survey

1. About the directed project of the second part of the course (you may select more than one option):
  (a) The freedom to design should stimulate creativity
  (b) I have doubts about the scope of the project
  (c) I prefer directed experiments in classes with specific criteria and procedures
  (d) I prefer tests to projects
2. Provide the name of your project.
3. Provide the summary of your project (problem, added value/benefit, results).
4. About the developed artifacts and components, select those that apply to your project (you can select more than one option):
  (a) VHDL test bench
  (b) VHDL code
  (c) Arduino Code
  (d) Python Code
  (e) Blynk Project in iOS or Android device
  (f) External Dashboard (e.g., JavaScript with HTML)
5. About the project development in 4 weeks, evaluate the project evolution considering the criteria:
  (a) Well-defined and successfully achieved goals
  (b) Well-defined goals, but partially achieved
  (c) Poorly defined goals, partial results
6. About the items below, rate their degree of importance to the project (necessary, very important, important, not important, it makes no difference):
  (a) Elevator pitch at the beginning of the class
  (b) OpenLab
  (c) Python Notebook
  (d) HomeLab
  (e) Previous experiments
  (f) IoT Platform
7. About remote collaboration with your team, provide a grade from 1 to 5 (with 5 being the best grade).
8. If you had to carry out the project again, what would you have done differently?
9. Describe the main lessons learned from the project.
